# Engineering the Optical Properties of CsPbBr_3_ Nanoplatelets through Cd^2+^ Doping

**DOI:** 10.3390/ma15217676

**Published:** 2022-11-01

**Authors:** Ivan D. Skurlov, Anastasiia V. Sokolova, Danila A. Tatarinov, Peter S. Parfenov, Danil A. Kurshanov, Azat O. Ismagilov, Aleksandra V. Koroleva, Denis V. Danilov, Evgeniy V. Zhizhin, Sergey V. Mikushev, Anton N. Tcypkin, Anatoly V. Fedorov, Aleksandr P. Litvin

**Affiliations:** 1 PhysNano Department, ITMO University, 197101 Saint Petersburg, Russia; 2 Laboratory of Quantum Processes and Measurements, ITMO University, 197101 Saint Petersburg, Russia; 3 Research Park, Saint Petersburg State University, 199034 Saint Petersburg, Russia

**Keywords:** perovskite, nanoplatelets, doping, photoluminescence, two-photon absorption

## Abstract

Lead halide perovskite nanoplatelets (NPls) attract significant attention due to their exceptional and tunable optical properties. Doping is a versatile strategy for modifying and improving the optical properties of colloidal nanostructures. However, the protocols for B-site doping have been rarely reported for 2D perovskite NPls. In this work, we investigated the post-synthetic treatment of CsPbBr_3_ NPls with different Cd^2+^ sources. We show that the interplay between Cd^2+^ precursor, NPl concentrations, and ligands determines the kinetics of the doping process. Optimization of the treatment allows for the boosting of linear and nonlinear optical properties of CsPbBr_3_ NPls via doping or/and surface passivation. At a moderate doping level, both the photoluminescence quantum yield and two-photon absorption cross section increase dramatically. The developed protocols of post-synthetic treatment with Cd^2+^ facilitate further utilization of perovskite NPls in nonlinear optics, photonics, and lightning.

## 1. Introduction

Lead halide perovskite nanoplatelets (NPls) are an emerging class of highly absorbing and strongly emitting optoelectronic nanomaterials [1,2]. Thanks to the quantum confinement effect, the ultranarrow photoluminescence (PL) band may be easily tuned by precise control of NPl thickness [3,4]. Different synthetic approaches were developed to synthesize both organic–inorganic hybrid and all-inorganic lead halide perovskite NPls, including hot-injection, ligand-assisted reprecipitation (LARP), ultrasonication, microwave-assisted, and solvothermal methods [5,6,7,8,9,10]. Pure emission color, high exciton binding energy, and uniform morphology make these nanostructures promising for light-emitting diodes (LEDs) [2,11,12]. It should be noted that 2D perovskite nanostructures attract much attention as candidates for numerous applications in the field of nonlinear optics and photonics [13,14].

B-site doping is a promising tool to improve both the optical properties and stability of lead halide perovskites. For instance, a partial substitution of Pb^2+^ atoms in CsPbI_3_ nanocrystals (NCs) with Sr^2+^ and Zn^2+^ aimed for the stabilization of a cubic α-phase [15,16]. Thermal and air stability of perovskite NCs were recently improved through Mn^2+^ and Cu^2+^ doping [17,18]. Interestingly, doping (alloying) the lead-free CsSnI_3_ perovskite with Pb^2+^ brings similar results [19]. Targeted doping has the most noticeable effect on the perovskite NCs photoluminescent (PL) properties. Particularly, the improvement of PL quantum yield and NC stability is essential for utilization of perovskite emitters in the ultraviolet-blue spectral range [20,21]. Another unique opportunity provided by B-site doping is a realization of a fundamentally different emission spectrum due to the introduction of such atoms as Mn^2+^, Yb^3+^, Er^3+^, etc. [22,23,24]. This strategy is useful both for obtaining NIR emitters and nanomaterials with a broad PL spectrum or with multiple emission bands. The numerous listed improvements that can be obtained through B-site doping have made this strategy widely researched. As a result, many effective strategies and protocols have emerged for obtaining doped NCs, both during synthesis and through post-synthetic processing [25,26,27].

In striking contrast, B-site doping has been rarely reported for 2D perovskite NPls and has been mainly focused on the introduction of manganese ions [28,29,30,31]. The peculiarities of the NPls’ growth, their ultrathin nature, and the increasing impact of a surface put forward fundamentally new requirements for the doping protocols development. Interestingly, it has been recently shown that doping may be utilized to control the CsPbBr_3_ NPls’ growth kinetics [32]. CsPbBr_3_ NPls can be considered as a perspective nanomaterial that may fill the perovskite-based LEDs low efficiency gap in the blue spectral region [33,34]. Therefore, the issues of improving their PL quantum yield, stability, and further PL band shift to the UV region of the spectrum are relevant. In the example of CsPbBr_3_ NCs, it was shown that the listed issues can be solved by doping with cadmium ions. Van der Stam et al. showed for the first time that post-synthetic treatment of CsPbBr_3_ NCs with Cd^2+^ precursor induces a blue shift of their absorption and PL bands due to the lattice contraction [35]. Xie et al. later revealed that treatment of CsPbBr_3_ NCs with CdX_2_ salts enhances their PL quantum yield [36]. We have recently presented that Cd^2+^ doping carried out during NCs synthesis leads not only to a change in the linear optical properties but also to a significant increase in the nonlinear optical response [37]. Zhao et al. demonstrated the performance of a white LED utilizing Cd^2+^-doped CsPbBr_3_ NCs grown inside a borosilicate glass as a green emitter [38].

These achievements motivated us to develop Cd^2+^ doping methods for CsPbBr_3_ NPls, aiming for their further utilization in nonlinear optics, optoelectronics, and lightning. In the current study, we demonstrate that the treatment of CsPbBr_3_ NPls with different Cd^2+^ precursors is an efficient tool to boost their linear and nonlinear optical properties via doping or/and surface passivation. We show that the interplay between the Cd^2+^ precursor, NPl concentrations, and ligands determines the doping process kinetics. We also show that the ultrathin nature of the NPls imposes restrictions on the doping level, and the highest quantum yield and the strongest nonlinear optical response can be achieved with a small number of inserted Cd^2+^ ions.

## 2. Materials and Methods

1-Octadecene (ODE, 90%, Merck, Rahway, NJ, USA), oleic acid (OA, 90%, Sigma-Aldrich), oleylamine (OlAm, 70%, Sigma-Aldrich, Burlington, MA, USA), octylamine (OctAm, 99%, Sigma-Aldrich), lead(II) bromide (PbBr_2_, ≥99.999%, Sigma-Aldrich), cesium carbonate (Cs_2_CO_3_, 99.9%, Sigma-Aldrich), cadmium(II) bromide tetrahydrate (CdBr_2_·H_2_O, 98%, Sigma-Aldrich), cadmium(II) acetate (Cd(OCOCH_3_)_2_, anhydrous, 99.995%, Sigma-Aldrich), and toluene (Vekton, Saint-Petersburg, Russia) were used as received without further purification.

CsPbBr_3_ NPls were synthesized and purified according to [31,39]. To obtain CdBr_2_ precursor, we added 18 mg CdBr_2_ to 2 mL of toluene with 15 µL of OlAm, degassed for 3 h at 80 °C, and then left stirred at 70 °C overnight. To obtain Cd(OCOCH_3_)_2_ precursor, we added 23 mg Cd(OCOCH_3_)_2_ to 2 mL of toluene with 15 µL of OlAm, stirred for 3 h at 100 °C, and then left stirred at 70 °C overnight.

Absorption and PL spectra were obtained by Shimadzu UV-3600 (Shimadzu Corporation, Kyoto, Japan) and Jasco FP-8200 (JASCO Deutschland GmbH, Pfungstadt, Germany), respectively. MicroTime 100 microscope (PicoQuant, Berlin, Germany) was used to record PL kinetics. 2PA-excited PL was investigated by a Coherent Astrella-USP ultrafast Ti:Sapphire amplifier (Coherent, Inc., Santa Clara, CA, USA) with a peak center at 800 nm, a pulse width of ~35 fs, 1 kHz repetition rate, and M266-IV (Standa Ltd., Vilnius, Lithuania) automated spectrograph. Escalab 250Xi (Thermo Fisher Scientific, Waltham, MA, USA) was used for XPS analysis. Zeiss Libra 200FE (Zeiss, Oberkochen, Germany) microscope and Solver Pro-M (NT-MDT, Moscow, Russia) microscope were used for transmission electron and atomic force microscopies, respectively.

## 3. Results

### 3.1. CsPbBr_3_ Nanoplatelets

**Figure 1:**
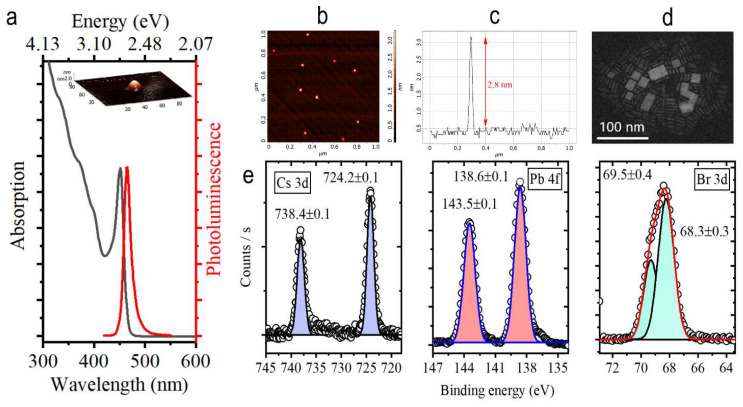
(**a**) Absorption and PL spectra of CsPbBr_3_ NPls. The inset shows a 3D AFM image. (**b**) AFM image of CsPbBr_3_ NPls and (**c**) the corresponding height profile. (**d**) HAADF-STEM image of CsPbBr_3_ NPls. (**e**) XPS spectra for Cs, Pb, and Br elements.

Figure 1a demonstrates the absorption and PL spectra of the synthesized CsPbBr_3_ NPls. The spectra show typical features of the NPls caused by 2D confinement, namely strong exciton absorption and a very narrow (FWHM = 16 nm) PL band. The PL quantum yield (PL QY) reached 19% after the synthesis and gradually decreased over time of storage (see Figure S1, Supplementary Materials). The morphology of the CsPbBr_3_ NPls was revealed using atomic force microscopy (AFM) and high-angle annular dark-field scanning transmission electron microscopy (HAADF-STEM). An AFM image of the NPls deposited onto a mica substrate is demonstrated in Figure 1b, while Figure 1c shows a corresponding height profile. The thickness of the NPLs was determined to be 2.8 nm, which was further confirmed by the analysis of vertically stacked NPls in HAADF-STEM images (Figure 1d and Figure S2, Supplementary Materials). The analysis of the lateral dimensions of ~150 NPls allowed us to estimate the average size of their side length, which is 17.5 ± 5.0 nm (see Figure S3, Supplementary Materials). Chemical composition of the synthesized NPls was confirmed by X-ray photoelectron spectroscopy (XPS). The XPS survey spectrum is demonstrated in Figure S4, and Figure 1e reveals the high-resolution XPS spectra for the Cs, Pb, and Br elements. The estimated positions of the corresponding peaks agree well with the literature data [40].

### 3.2. Doping with CdBr_2_

**Figure 2:**
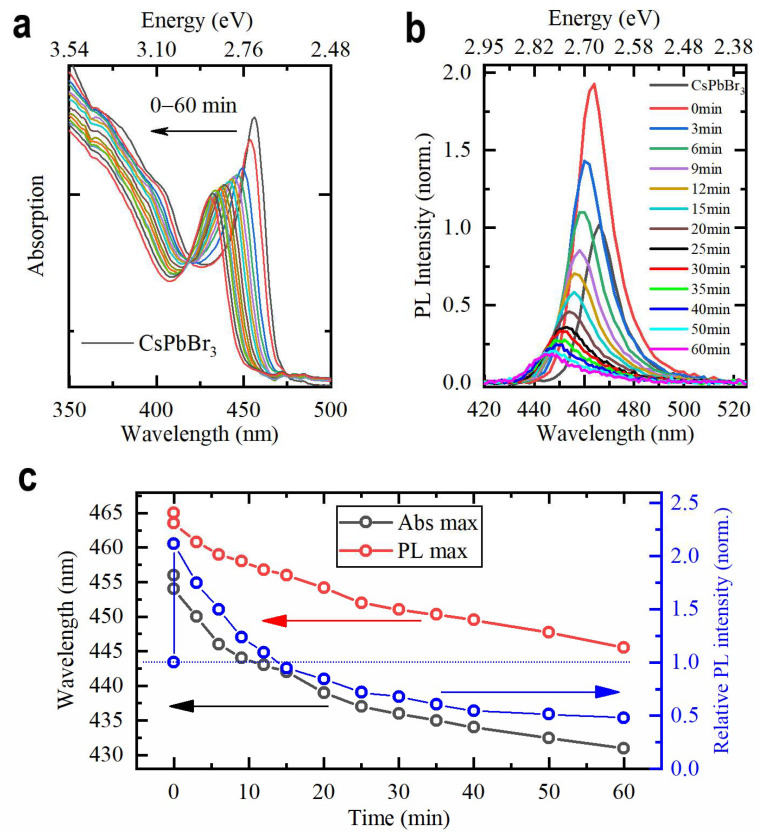
Evolution of (**a**) absorption and (**b**) PL spectrum under Cd^2+^ doping. (**c**) Evolution of absorption and PL peak positions and relative PL intensity.

In the case of using cadmium bromide as a Cd^2+^ precursor, the doping efficiency depends on the amount of added precursor, the amount and concentration of perovskite NPls, and the ligand density. The gradual process of doping can be directly observed over time for nonconcentrated solutions, with the doping rate being determined by the amount of precursor. Figure 2a,b shows the evolution of absorption and PL spectra of the NPls when 25 µL of CdBr_2_ precursor was added to 3 mL of NPl toluene solution with 0.15 optical density at 400 nm (NPl concentration is ~10^−8^ M). The dynamics of the PL and absorption peak positions over time are shown in Figure 2c. The absorption spectra reveal gradual blue shifts, indicating the bandgap increase. This widening of the bandgap is due to a lattice contraction induced by the progressive replacement of a Pb^2+^ cation with a Cd^2+^ cation with a smaller ionic radius [35]. In general, the PL peak position follows this trend. Figure S5 shows the evolution of PL peak position (in eV) and FWHM under Cd^2+^ doping. The PL maximum gradually shifts by 117 meV to the high-energy region of the spectrum, indicating a significant increase in the PL energy. It is worth noting that the insertion of Cd^2+^ ions is accompanied by a slight increase in Stokes shift, which originates from a stronger shift of an absorption band as compared to a PL band. We may speculate that the increase in Stokes shift is attributed to the lattice contraction, since an absorption spectrum is more sensitive to compression [41].

**Figure 3:**
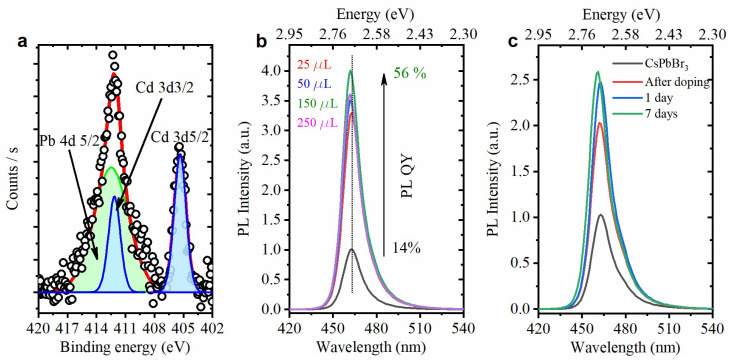
(**a**) XPS spectrum of Cd 3d peaks taken for the Cd^2+^-doped NPls with PL peak position at 445 nm. (**b**) PL spectra of NPls after adding various amounts of CdBr_2_ with the increased amount of OlAm. (**c**) PL spectra of NPls after adding 20 µL of the precursor with octylamine before and after doping, as well as during seven days’ storage.

Blue circles in Figure 2c show the evolution of relative PL intensity (determined as an integrated PL intensity divided by an optical density at the excitation wavelength and normalized to the value obtained for the initial solution). Immediately after the addition of the Cd^2+^ precursor, a significant growth in PL efficiency occurs, which may be attributed to the efficient surface passivation. It has recently been proven that the passivation of CsPbX_3_ perovskite NCs with CdX_2_ leads to the PL QY enhancement due to the filling of the Pb−X vacancies with Cd^2+^ and X^−^ ions [36]. During further Cd^2+^ doping, relative PL intensity decreases gradually and reaches a plateau at about 0.5 from its original value. In order to confirm that cadmium ions are incorporated into the NPls, we performed an XPS analysis for a sample with a PL peak position at a wavelength of 445 nm. Figure 3a shows the spectrum for Cd 3d peaks, and the estimated peak positions of 405.3 ± 0.1 eV and 412.1 ± 0.1 eV for the Cd 3d5/2 and Cd 3d3/2, respectively, are in good agreement with data obtained in [37,38]. The NPls’ chemical composition CsPb_0.84_Cd_0.16_Br_3_ was calculated using the Cd 3d5/2 spectrum.

It was found that the ligand concentration in the precursor solution significantly affects the NPl doping. Increasing the OlAm amount in the precursor by a factor of 15 considerably inhibits doping; however, the PL QY significantly grows in this case. Figure 3b shows the PL spectra of NPls after adding various amounts of the cadmium precursor with the increased OlAm amount. The PL QY reaches an impressive value of 56%, which, however, is accompanied by only a slight shift of the PL band. A similar situation is observed when OlAm is replaced by octylamine in the precursor. Figure 3c demonstrates the NPls’ PL spectra after adding 20 µL of the precursor with octylamine. Similarly to the procedure with OlAm, we observe a significant increase in the PL intensity, which persists for at least seven days’ storage.

Alternatively, the doping was tested on the solutions with NPl concentration increased by ten times (See Figure S6, Supplementary Materials). In this case, a growth in the PL QY is also observed, while there is no deterioration in the PL properties of the NPls with an increase in the Cd^2+^ amount, as was observed for diluted solutions (Figure 2c). It should also be noted that in this case, it is possible to achieve a smaller shift of the PL band position, indicating less Cd^2+^ doping. The interpretation of the results obtained under various doping regimes is in agreement with existing hypotheses, according to which doping occurs due to the introduction of cadmium bromide as a whole unit [35]. Upon dilution, the decrease in PL QY and distortion of the PL spectrum are observed (see Figure S7, Supplementary Materials). Dilution of the NPl solution promotes the formation of a larger number of surface defects, including halide vacancies, which can be filled then with cadmium bromide, followed by the possible migration of cations. Moreover, the NPls’ ultrathin nature significantly contributes to doping. The introduction of a large number of ions with a smaller ionic radius adds to the destabilization of the NPl structure and, as a consequence, to a decrease in PL efficiency. On the contrary, at a low doping level surface passivation occurs at the same time, leading to a significant increase in PL QY.

### 3.3. Doping with Cd(OCOCH_3_)_2_

**Figure 4:**
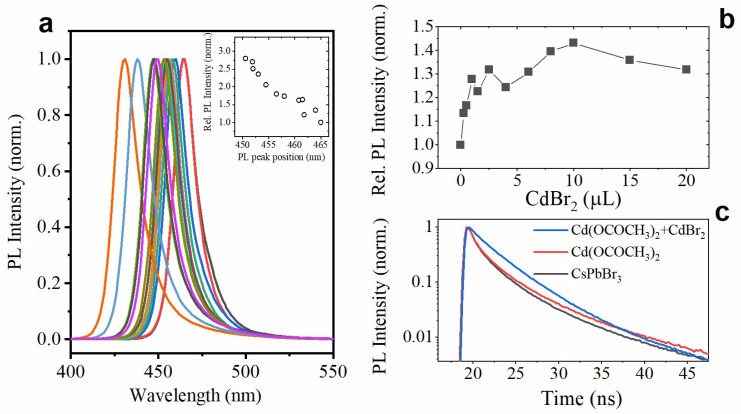
(**a**) PL spectra of Cd^2+^-doped NPls with various amounts of Cd(OCOCH_3_)_2_ precursor solution. The inset shows the dependence of PL QY on the PL peak position. (**b**) Relative PL intensity of the NPls doped with Cd(OCOCH_3_)_2_ (PL peak position at 455 nm) after the addition of various amounts of CdBr_2_. (**c**) PL decay curves taken for the undoped NPls (black line), doped with Cd(OCOCH_3_)_2_ (red line), and CdBr_2_ treated after Cd(OCOCH_3_)_2_ doping (blue line).

Doping was additionally realized using Cd(OCOCH_3_)_2_ as a Cd^2+^ source. In this case, 1 µL of Cd(OCOCH_3_)_2_ was mixed with 1 µL of OA, 1 µL OlAm, and 10 µL of toluene for the further use. Then, a specified amount (1–13 µL) of this precursor was added to the colloidal solution of NPls. As shown in Figure 4a, increasing the amount of an added precursor causes a smooth shift of the PL band to the blue region of the spectrum, which indicates a gradual increase in the number of introduced cadmium ions. The shift is accompanied by growth in relative PL intensity, as shown in the inset of Figure 4a.

It seems important to notice that the NPls doped with the use of Cd(OCOCH_3_)_2_ can be further passivated with CdBr_2_. As an example, Cd^2+^-doped NPls with a PL peak position at 452 nm were treated with a successively increasing amount of CdBr_2_ precursor. Figure 4b demonstrates the dependence of the NPl solution’s relative PL intensity on the amount of CdBr_2_ precursor. Additional treatment of the Cd^2+^-doped NPls with CdBr_2_ allows for further improvement of their luminescent properties due to the extra passivation of their surface, which is confirmed by studying the PL kinetics (see Figure 4c). While doping with Cd(OCOCH_3_)_2_ has a little effect on PL kinetics (intensity-weighted average PL lifetime increases from 3.5 ns to 3.6 ns), further treatment with CdBr_2_ changes the shape of the PL decay curve and induces a pronounced growth of an average PL lifetime up to 4.6 ns. Thus, a double-step treatment with different Cd^2+^ precursors allows for both PL wavelength tuning and surface passivation.

We may underline the following observations to summarize the results of the proposed CsPbBr_3_ NPls doping protocols with Cd^2+^ ions. Too high a rate of doping is harmful to NPls’ structural stability and can be achieved through the use of CdBr_2_ in diluted solution. Using highly concentrated NPl solutions or introducing additional ligands allows for slowing down the doping rate and keeping a strong surface passivation effect. In this case, however, the wavelength tunability is insufficient. The doping with Cd(OCOCH_3_)_2_ is a more suitable choice for tuning the position of the NPls emission. Further passivation may be achieved via a second stage with CdBr_2_, providing a spectral shift, a high PL quantum yield, and stability, as listed in Table 1.

### 3.4. Two-Photon-Induced Photoluminescence

**Figure 5:**
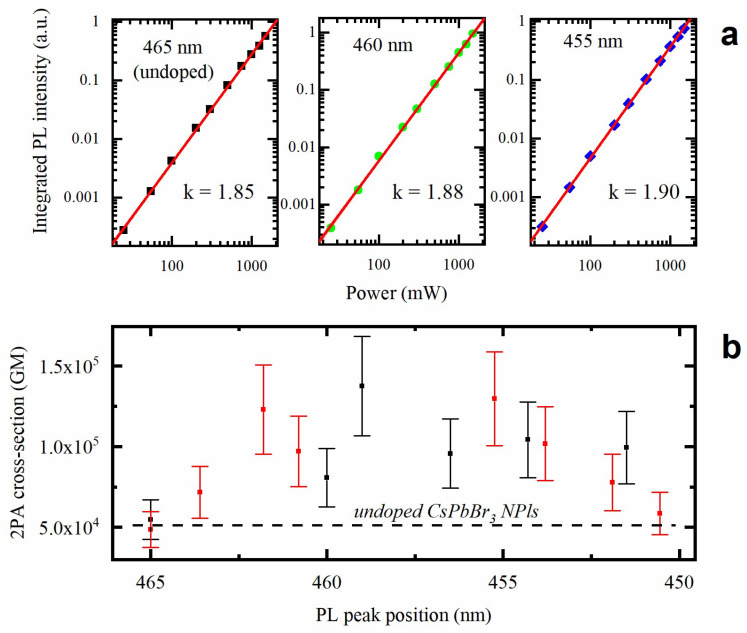
(**a**) The log–log plots of the integrated PL intensity vs. excitation power obtained for the parent and Cd^2+^-doped samples. (**b**) The dependence of the 2PA absorption cross section on emission peak position for two batches of Cd^2+^-doped samples. The dashed line indicates the value obtained for the undoped sample.

To investigate the influence of doping with Cd(OCOCH_3_)_2_ on the NPls’ nonlinear optical responses, we studied their PL when excited via the two-photon absorption (2PA) process. Figure 5a demonstrates the log–log plots of the integrated PL intensity vs. excitation power. The dependences obtained for both the doped and the parent samples are well-described by a linear function with a slope close to 2, which corresponds to a 2PA-excited emission. The 2PA absorption cross section is an important figure of merit to compare the nonlinear optical processes in different materials. 2PA cross sections for the parent and Cd^2+^-doped NPls were calculated using fluorescein as a standard [42]. Figure 5b shows the dependence of the 2PA absorption cross section on the emission peak position for two batches of samples. One can see that NPls possessing the PL maximum in the range 455–460 nm demonstrate the strongest nonlinear absorption. Assuming a linear dependence of the PL peak position on the doping ratio [35], we can conclude that the replacement of 4−8% of the Pb^2+^ ions with Cd^2+^ ions in a B-site position allows for the achievement of superior nonlinear optical properties. Such a small fraction of doped Cd^2+^ ions induces a strong enhancement (more than 2.5 times) of nonlinear absorption. This observation is in a good agreement with experimental and theoretical studies of doped perovskite nanocrystals [35,37,43]. Ahmed et al. recently concluded that the insertion of even a small fraction of doping ions results in the local lattice periodicity breaking, which leads to a charge carrier localization [43]. At a higher level of doping, 2PA starts to decline, which may be attributed to the structural instability of the doped NPls under the intense laser radiation. Indeed, the 2PA-excited PL spectrum of the slightly doped NPls is well-superimposed on the one-photon-excited PL spectrum (see Figure S8, Supplementary Materials). On the contrary, for heavily doped NPls, a shoulder appears on the low-energy side in the spectrum of 2PA-excited PL, which can be associated with the relaxation through the emerging surface and structural defects.

## 4. Conclusions

To sum up, we have developed the methods for the CsPbBr_3_ NPls post-synthetic doping with Cd^2+^ ions. We found that the interplay between Cd^2+^ precursor, NPl concentrations, and ligands determines the kinetics of the doping process. We showed that a careful choice of doping conditions allows for the achievement of significant improvement in NPl optical properties. Additionally, we developed a two-step doping protocol that allows for fine emission wavelength tuning, boosts PL quantum yield, and preserves the NPls’ stability. We also demonstrated that the replacement of 4–8% of the Pb^2+^ ions with Cd^2+^ ions in a B-site position induces a strong enhancement of the nonlinear optical response; particularly, the two-photon absorption cross section is increased by 2.5 times. We showed that the doping level is limited by the structural instability of ultrathin NPls occurring due to the introduction of a large number of dopants with a smaller ionic radius. These findings reveal more opportunities for the use of lead halide NPls in optoelectronic devices, as well as for the development of methods for doping perovskite nanostructures.

## Figures and Tables

**Table 1:** The summary of CsPbBr_3_ NPls doping with Cd^2+^.

Doping Type	Doping Rate	PL Peak Position Tuning	Stability	Passivation
CdBr_2_, diluted	High	Yes	Low	Moderate
CdBr_2_, concentrated	Low	No	High	High
CdBr_2_, ligands	Low	No	High	High
Cd(OCOCH_3_)_2_	Moderate	Yes	Moderate	Moderate
Double-step	Moderate	Yes	High	High

## Data Availability

Not applicable.

## Supplementary Materials

The following supporting information can be downloaded at: https://www.mdpi.com/article/10.3390/ma15217676/s1, Figure S1: CsPbBr_3_ NPls PL QY shift over time of storage; Figure S2: HAADF-STEM images of the CsPbBr_3_ NPls; Figure S3: An analysis of lateral dimensions of the CsPbBr_3_ NPls; Figure S4: The XPS survey obtained for the CsPbBr_3_ NPls; Figure S5: Evolution of PL peak position and FWHM under Cd^2+^ doping. Figure S6: The shift of PL spectra of CsPbBr_3_ NPls upon Cd^2+^ doping in concentrated solutions. The inset shows the relative PL intensity of the solutions obtained using different CdBr_2_ precursor amount; Figure S7: PL spectra of CsPbBr_3_ NPl solutions with optical densities of 0.15 and 0.03 (at 400 nm). The inset shows the normalized dependence of PL QY on the optical density of the NPl solution; Figure S8: PL spectra for two Cd^2+^-doped samples with a different Cd^2+^ amount (emission wavelengths are 447 nm and 45 nm, respectively) obtained under one-photon absorption (1PA) and two-photon absorption (2PA) excitation.
