# Supplementary material for: Engineering the Optical Properties of CsPbBr3 Nanoplatelets through Cd2+ Doping
Source: Materials (Basel). 2022 Nov 1;15(21):7676. doi: 10.3390/ma15217676 (PMC9657966; doi:10.3390/ma15217676)
Supplement: Supplementary file 1 [file materials-15-07676-s001.zip › materials-2000827-supplementary.pdf]

materials

MDPI

Supplementary Information

Engineering the Optical Properties of $CsPbBr_3$ Nanoplatelets through Cd^{2+} Doping

Ivan D. Skurlov [1], Anastasiia V. Sokolova [1], Danila A. Tatarinov [1], Peter S. Parfenov [1], Danil A. Kurshanov [1], Azat O. Ismagilov [2], Aleksandra V. Koroleva [3], Denis V. Danilov [3], Evgeniy V. Zhizhin [3], Sergey V. Mikushev [3], Anton N. Tcypkin [2], Anatoly V. Fedorov [1] and Aleksandr P. Litvin [1,2,*]

[1] PhysNano Department, ITMO University, 197101 Saint Petersburg, Russia
[2] Laboratory of Quantum Processes and Measurements, ITMO University, 197101 Saint Petersburg, Russia
[3] Research Park, Saint Petersburg State University, 199034 Saint Petersburg, Russia
* Correspondence: litvin@itmo.ru

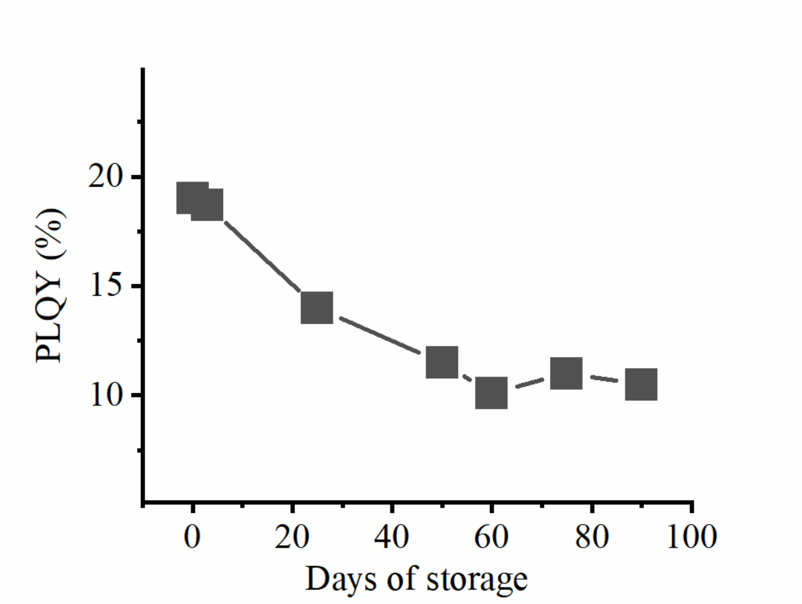

Figure S1. $CsPbBr_3$ NPls PL QY shift over time of storage.

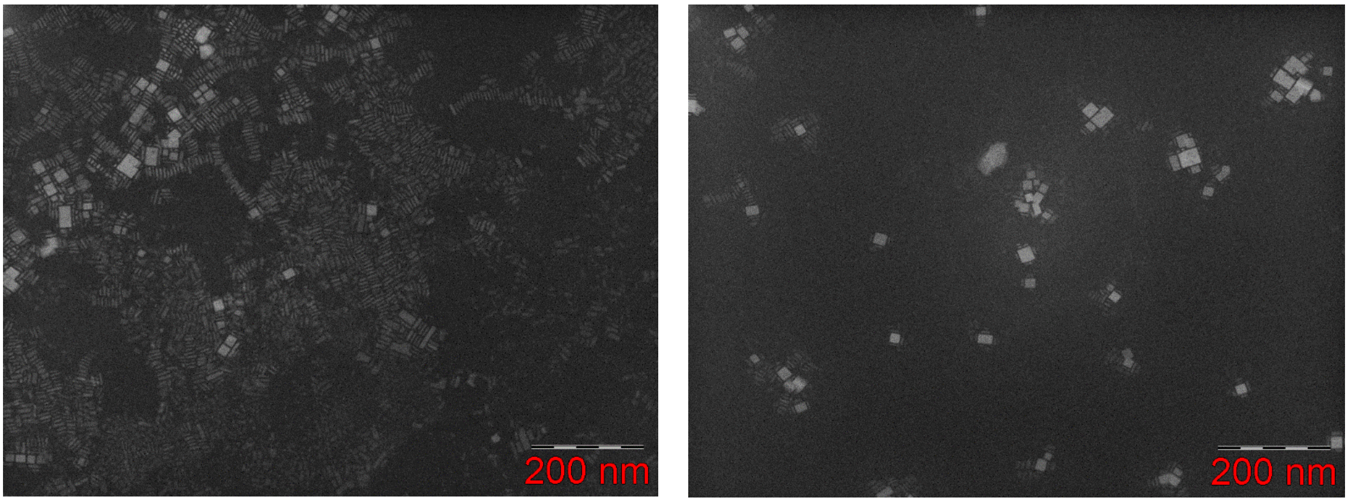

Figure S2. HAADF-STEM images of the $CsPbBr_3$ NPls .

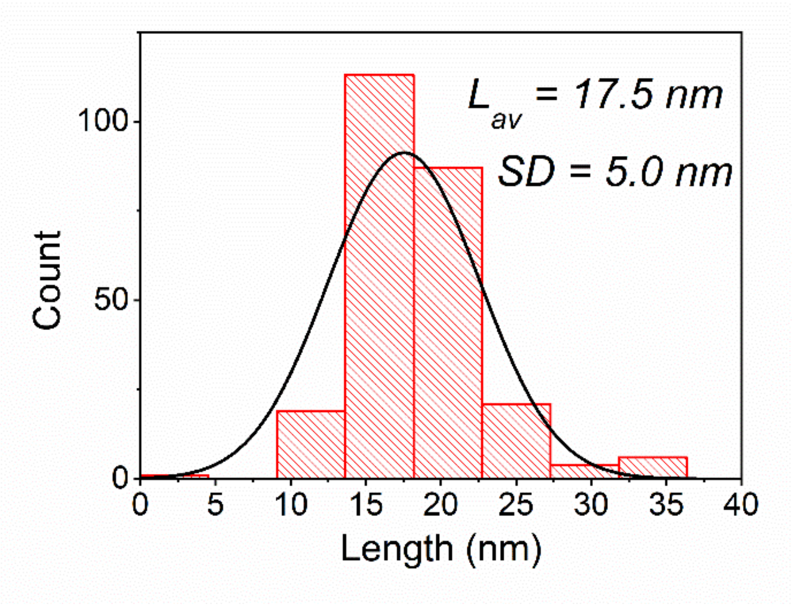

Figure S3. An analysis of lateral dimensions of the $CsPbBr_3$ NPls.

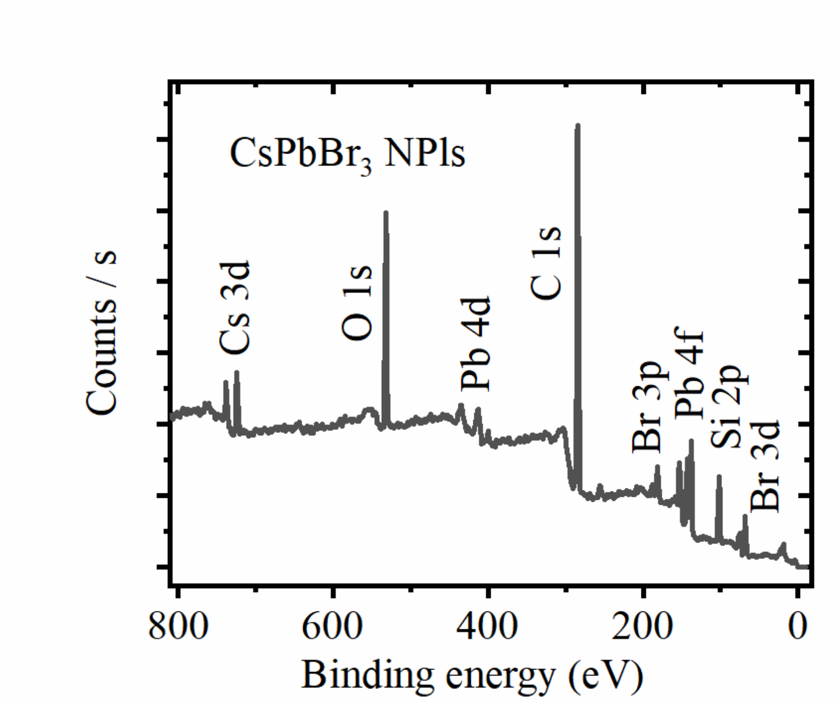

Figure S4. The XPS survey obtained for the $CsPbBr_3$ NPls.

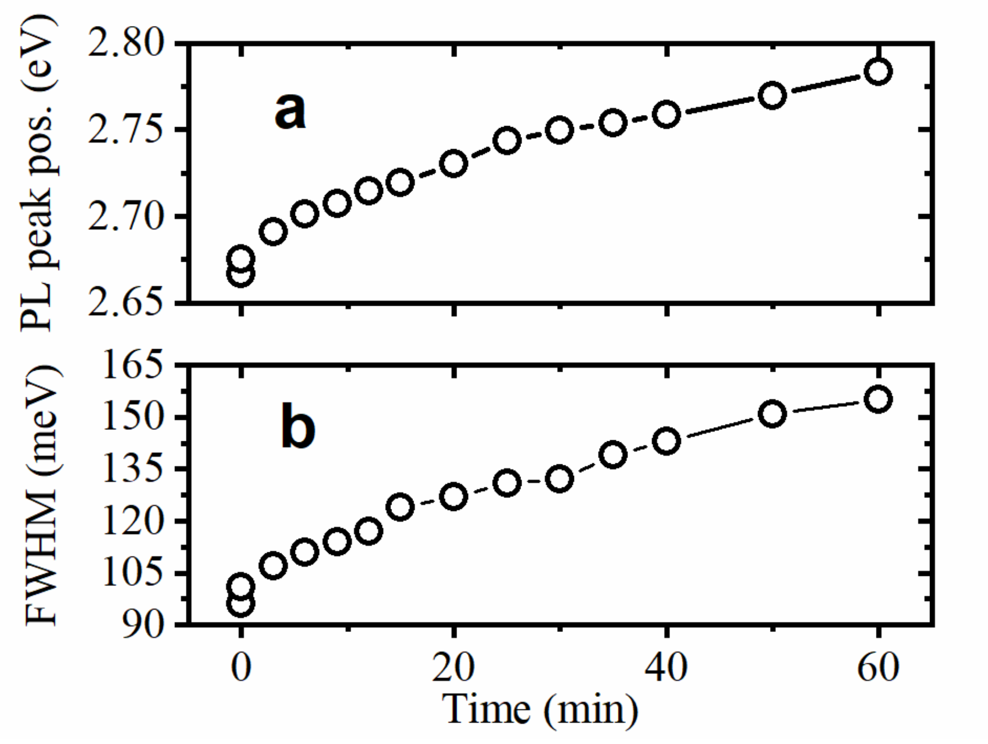

Figure S5. Evolution of (**a**) PL peak position and (**b**) FWHM under Cd^{2+} doping.

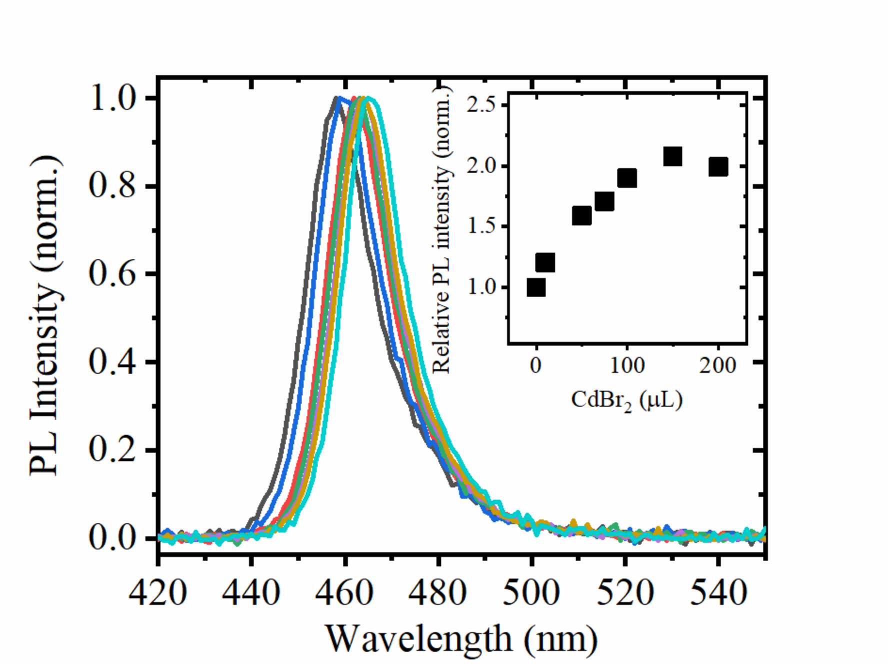

Figure S6. The shift of PL spectra of $CsPbBr_3$ NPls upon Cd^{2+} doping in concentrated solutions. The inset shows the relative PL intensity of the solutions obtained using different $CdBr_2$ precursor amount.

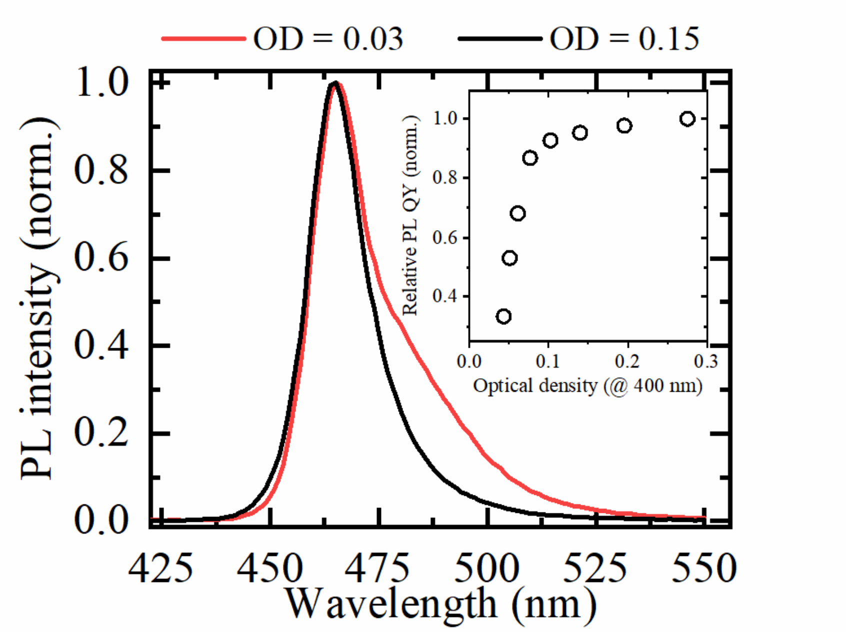

Figure S7. PL spectra of $CsPbBr_3$ NPls solutions with optical densities of 0.15 and 0.03 (at 400 nm). The inset shows the normalized dependence of PL QY on the optical density of the NPls solution.

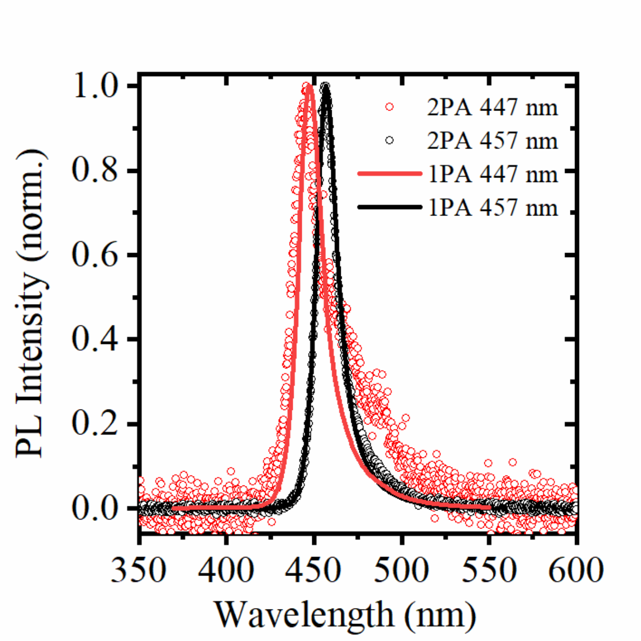

Figure S8. PL spectra for two Cd^{2+}-doped samples with a different Cd^{2+} amount (emission wavelengths are 447 nm and 45 nm, respectively) obtained under one-photon absorption (1PA) and two-photon absorption (2PA) excitation.
